# High-Resolution Vessel Wall MRI of Endovascularly Treated Intracranial Aneurysms

**DOI:** 10.3390/tomography8010025

**Published:** 2022-02-01

**Authors:** Łukasz Zwarzany, Mateusz Owsiak, Ernest Tyburski, Wojciech Poncyljusz

**Affiliations:** 1 Department of Diagnostic Imaging and Interventional Radiology, Pomeranian Medical University in Szczecin, Unii Lubelskiej 1, 71-252 Szczecin, Poland; mateusz.owsiak@pum.edu.pl (M.O.); wojciech.poncyljusz@pum.edu.pl (W.P.); 2 Department of Health Psychology, Pomeranian Medical University in Szczecin, Broniewskiego 26, 71-460 Szczecin, Poland; ernest.tyburski@pum.edu.pl

**Keywords:** intracranial aneurysm, vessel wall imaging, MRI, endovascular treatment

## Abstract

(1) Background: The aim of this study was to determine the frequency and the pattern of post-procedural intracranial aneurysm contrast enhancement on high-resolution vessel wall magnetic resonance imaging (HR-VW MRI). We investigated the possible association between this imaging finding and factors such as time elapsed since embolization or aneurysm occlusion grade on baseline and follow-up imaging. (2) Methods: Consecutive patients presenting for follow-up after endovascular treatment of intracranial aneurysms were included. HR-VW MRI was acquired and interpreted independently by two radiologists. (3) Results: This study included 40 aneurysms in 39 patients. Contrast enhancement was detected in 30 (75%) aneurysms. It was peripheral in 12 (30.0%), central in 9 (22.5%), and both peripheral and central in 9 (22.5%) aneurysms. The statistical analysis did not reveal any relationship between follow-up period and the presence of contrast enhancement (*p* = 0.277). There were no statistically significant differences in the frequency of contrast enhancement between aneurysms with total occlusion and those with remnant flow on follow-up MR angiography (*p* = 0.850) nor between aneurysms with different interval changes in the aneurysm occlusion grade (*p* = 0.536). Multivariate analysis did not demonstrate aneurysm size, ruptured aneurysm status, nor initial complete aneurysm occlusion to be a predictor of contrast enhancement (*p* = 0.080). (4) Conclusions: Post-procedural aneurysm contrast enhancement is a common imaging finding on HR-VW MRI. The clinical utility of this imaging finding, especially in the prediction of aneurysm recurrence, seems limited. The results of our study do not support routine use of HR-VW MRI in the follow-up of patients after endovascular treatment of intracranial aneurysms.

## 1. Introduction

Following endovascular intracranial aneurysm repair, patients are routinely followed up with imaging studies as recanalization occurs in as many as 20% of cases [1]. Although digital subtraction angiography (DSA) remains the gold standard for the identification of residual or recurrent aneurysm, magnetic resonance angiography (MRA) is widely used due to its non-invasive nature and good diagnostic performance [2].

High-resolution vessel wall magnetic resonance imaging (HR-VW MRI) provides new possibilities for the evaluation of the intracranial vasculature [3]. The number of studies on the use of HR-VW MRI in patients with intracranial aneurysms is constantly growing. Aneurysm wall enhancement (AWE) on HR-VW MRI may identify ruptured aneurysm in patients with aneurysmal subarachnoid hemorrhage (aSAH) and multiple aneurysms [4,5,6,7,8]. It is also presumed to be an imaging marker of aneurysm instability, indicating lesions with higher risk of rupture [9,10,11,12,13,14,15,16,17,18,19,20,21,22,23,24,25,26]. This hypothesis is consistent with the results of our previous study, in which we have shown a statistically significant association between AWE and conventional risk factors for aneurysm rupture [27]. Similar to our previous study, most of the published literature on the use of HR-VW MRI in patients with intracranial aneurysms focuses on unruptured intracranial aneurysms (UIA) and determining whether AWE is an imaging marker of aneurysm instability. This trend might be explained by the fact that such a marker is highly desirable as the management of UIA remains challenging.

However, the management of patients after intracranial aneurysm repair also remains an important medical issue. The number of such patients is constantly growing, which results in an increasing number of follow-up imaging studies. These studies constitute a significant burden for the diagnostic imaging providers. Unfortunately, we lack an imaging marker of a definitive aneurysm healing completion. If such a marker of stable aneurysm occlusion existed, some patients could be followed up less frequently.

Thus, we believe that research on the use of HR-VW MRI in patients with intracranial aneurysm should not be restricted to AWE and prediction of aneurysm rupture but other applications of this imaging tool should also be investigated. While there is growing evidence for the use of HR-VW MRI in therapeutic decision making in patients with UIA, the possible clinical significance of post-procedural aneurysm contrast enhancement has not been widely studied, with only a few papers published [28,29,30].

As the data on the use of HR-VW MRI in the follow-up of patients after intracranial aneurysm repair are scarce, we decided to further investigate this issue. The goal of our present study was to evaluate the occurrence and the pattern of contrast enhancement of endovascularly treated aneurysms on HR-VW MRI. Furthermore, we investigated the possible association between aneurysm contrast enhancement and factors such as time elapsed since embolization, aneurysm size, ruptured aneurysm status, and aneurysm occlusion grade on baseline and follow-up imaging.

## 2. Materials and Methods

### 2.1. Patients and Study Design

The local ethics committee approved this study (KB-0012/112/19). We prospectively recruited consecutive adult patients who presented at our institution between January 2020 and June 2021 for routine imaging follow-up after coiling or stent-assisted coiling of intracranial aneurysm and underwent HR-VW MRI. Written informed consent was obtained from all patients prior to imaging.

### 2.2. HR-VW MRI Protocol

HR VW-MRI was performed on a 3.0T MRI system (Signa Pioneer; GE Healthcare, Milwaukee, WI, USA) using a 21-channel head/neck coil. The imaging protocol included three-dimensional (3D) time of flight MR angiography (TOF MRA), and pre- and postcontrast 3D fast spin-echo (FSE) T1-weighted (T1W) sequence with variable refocusing flip angles (CUBE; GE Healthcare). The acquisition parameters of 3D FSE T1W sequence were as follows: repetition time (TR)/echo time (TE) = 604 ms/minimum; field of view (FOV) = 180 × 180 mm; matrix = 224 × 224; bandwidth = 50.0 kHz; echo train length (ETL) = 30; spatial resolution = 0.8 × 0.8 × 0.8 mm (interpolated to 0.8 × 0.8 × 0.4 mm); number of excitations (NEX) = 4. Gadoterate meglumine (Dotarem; Guerbet LLC, Villepinte, France) was administered intravenously (0.1 mmol/kg), and a 3D FSE T1W sequence was repeated with a 5 min delay after contrast agent injection.

### 2.3. HR-VW MRI Analysis

HR-VW MRI images were reviewed independently by two radiologists, who determined the presence of post-procedural aneurysm contrast enhancement by comparing pre- and postcontrast images. The precontrast images were carefully assessed for the presence of intra-aneurysmal thrombus so as not to misinterpret it as an area of contrast enhancement. For each aneurysm, reviewers were asked to classify the contrast enhancement pattern into one of the four categories: no contrast enhancement, peripheral contrast enhancement, central contrast enhancement, or peripheral and central contrast enhancement. Aneurysm contrast enhancement was evaluated as peripheral if it was restricted to the aneurysm wall or to the part of the intra-aneurysmal thrombus directly adjacent to it. Peripheral contrast enhancement could involve a part or the entire circumference of the aneurysm. If peripheral contrast enhancement involved only the part of the aneurysm adjacent to the cavernous sinus, it was required to be definitely stronger than the enhancement of the cavernous sinus. Central aneurysm contrast enhancement was defined as the enhancement limited to the central part of the intra-aneurysmal thrombus, with no contact to the aneurysm wall. It also included the intra-aneurysmal thrombus enhancement around the aneurysm remnant. In the peripheral and central contrast enhancement pattern, both types of enhancement had to be present.

**Figure 1:**
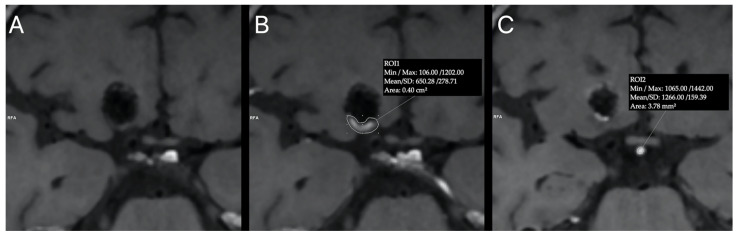
Example of region of interest (ROI) placement for quantitative analysis of post-procedural aneurysm contrast enhancement intensity. Precontrast (**A**) and postcontrast (**B**,**C**) HR-VW MRI of right internal carotid artery aneurysm treated with stent-assisted coiling. ROI drawn manually around the area of peripheral aneurysm contrast enhancement (**B**). Second ROI positioned within the pituitary stalk (**C**).

Furthermore, for the aneurysms which, in the subjective assessment of the readers, showed contrast enhancement, a quantitative assessment of contrast enhancement intensity was performed. Using the postcontrast HR-VW MRI images, the aneurysm-to-pituitary stalk contrast enhancement ratio was determined. It was calculated as the ratio of the maximal signal intensity in a manually drawn region of interest (ROI) containing aneurysm contrast enhancement (either peripheral or central) over the maximal signal intensity in a manually drawn ROI within the pituitary stalk (Figure 1). Reviewers assessed the aneurysm occlusion grade on TOF MRA images using the Raymond Roy Occlusion Classification (RROC) [31]. In this study, aneurysm recurrence was defined as any progression on the RROC in comparison with the immediate post-procedural DSA images. Disagreements between reviewers were resolved by consensus.

### 2.4. Post-Procedural Angiographic Findings

Digital subtraction angiography images acquired during the embolization procedures were retrieved from the local picture archiving and communication system (PACS). The same radiologists who interpreted HR VW-MRI also evaluated DSA images. Anonymized DSA images were given to the reviewers in a random order, at least 4 weeks after the interpretation of HR VW-MRI was completed. Aneurysm location was determined, aneurysm size (defined as the largest aneurysm diameter in any direction) was measured, and initial aneurysm occlusion grade was assessed using RROC. DSA images were evaluated independently by the reviewers and, in the case of disagreement, the final decision was made by consensus. Other clinical and demographic data, such as aneurysm status (ruptured or unruptured) and time interval between embolization and HR-VW MRI, were obtained from the medical records.

### 2.5. Statistical Analysis

Statistical analysis of the results was carried out using the IBM SPSS 26 Statistical package (IBM Corp, Redmont, VA, USA). Continuous variables are presented as mean (M) and standard deviation (SD), while nominal variables are presented as number (n) and percent (%). The normality of the distribution of continuous variables was tested with the Shapiro–Wilk test. The differences in proportions of aneurysms with contrast enhancement between aneurysms with complete occlusion and those with remnant flow on follow-up MR angiography and between aneurysms with different interval changes in the RROC, and between aneurysms treated with coiling and stent-assisted coiling were analyzed using the chi-square test for cross-tabulation with Yates continuity correction for 2 × 2. The chi-square test for cross-tabulation was also used to evaluate the differences in aneurysm occlusion grade between aneurysms treated with coiling and stent-assisted coiling. Simple linear regression was performed to identify the relationship between time elapsed since embolization and aneurysm contrast enhancement intensity. Simple logistic regression was used to explore the relationship between time elapsed since embolization and the presence of aneurysm contrast enhancement. Furthermore, multivariate logistic regression (backward elimination, the Wald chi-square test) was further performed to identify predictors of aneurysm contrast enhancement (with the Hosmer–Lemeshow test for evaluating the goodness of fit of logistic regression models). For multivariate analysis, we assumed ten observations per one variable as a minimum according to Long [32]. The alpha criterion level was set at 0.05 in all statistical analyses. Kendall’s W (for ordinal variables) and Cohen’s Kappa (for nominal variables) were calculated to measure the agreement between two independent readers. The effect sizes of Kendall’s W and Kappa correlations were adopted based on Cohen et al. and McHugh et al., respectively [33,34].

## 3. Results

### 3.1. Patients and Aneurysms Characteristics

Thirty-nine patients with 40 aneurysms were included in this study. The mean age of the patients was 52.87 (range, 30–72) and 32 (82.1%) of them were females.

The mean aneurysm size was 6.84 mm (SD = 4.22; range: 3–24 mm). Among them, 11 (27.5%) were ruptured and 29 (72.5%) were unruptured. Sixteen (40.0%) aneurysms were located in the internal carotid artery, nine (22.5%) in the middle cerebral artery, eight (20.0%) in the anterior cerebral arteries (including the anterior cerebral artery, the anterior communicating artery, and the pericallosal artery), five (12.5%) in the posterior communicating artery, and two (5.0%) in the posterior circulation (including the vertebral artery, the basilar artery, the cerebellar arteries, and the posterior cerebral artery). Patient and aneurysm characteristics are summarized in Table 1.

### 3.2. Procedural Details and Immediate Angiographic Outcome

Fifteen (37.5%) aneurysms were treated with coiling, and the other 25 (62.5%) with stent-assisted coiling. Bare platinum coils were used in all cases. In patients treated with stent-assisted coiling, the LVIS or LVIS Jr. stent (MicroVention Terumo, Tustin, CA, USA) was implanted. Immediate post-procedural angiography revealed complete occlusion in 32 (80.0%) aneurysms, neck remnant in 8 (20.0%) aneurysms, and aneurysm remnant in 0 (0.0%) aneurysms. There was no difference in immediate angiographic outcome between aneurysms treated with coiling and stent-assisted coiling (Table 2; x2 = 0.000; *p* = 1.000).

### 3.3. Follow-Up MR Angiography

The mean time elapsed between embolization and follow-up MR angiography was 16.11 months (SD = 15.98; range, 0.07–67 months). Follow-up MRA demonstrated complete aneurysm occlusion in 25 (62.5%) aneurysms, neck remnant in 10 (25.0%) aneurysms, and aneurysm remnant in 5 (12.5%) aneurysms. There was a statistically significant difference in aneurysm occlusion grade on follow-up MR angiography between aneurysms treated with coiling and stent-assisted coiling (Table 2; x2 = 9.963; *p* = 0.007).

### 3.4. Post-Procedural Aneurysm Contrast Enhancement on HR-VW MRI

**Figure 2:**
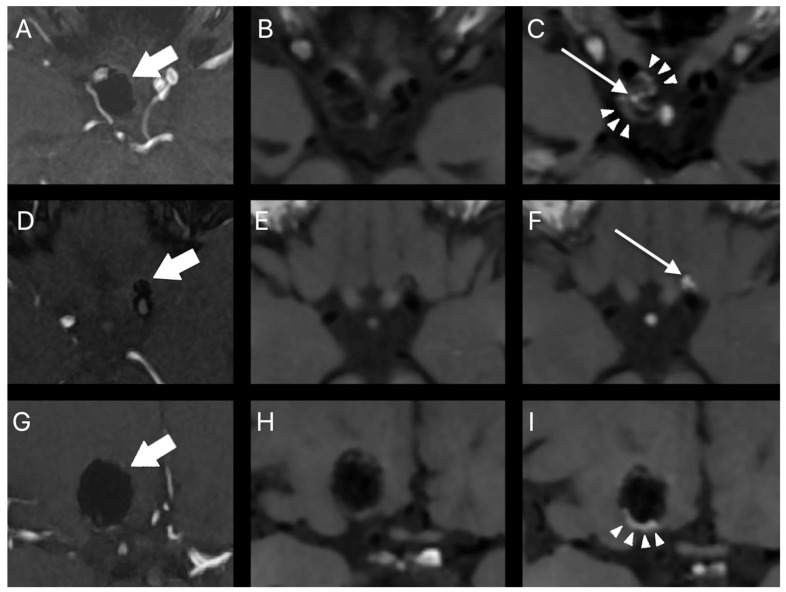
Representative cases of post-procedural aneurysm contrast enhancement on HR-VW MRI. Follow-up TOF MRA (**A**,**D**,**G**) and corresponding precontrast (**B**,**E**,**H**) and postcontrast (**C**,**F**,**I**) HR-VR MRI. Right internal carotid artery aneurysm treated with stent-assisted coiling (thick arrow) (**A**–**C**). There is no residual flow in the aneurysm cavity. Postcontrast HR-VW MRI shows contrast enhancement at the aneurysm periphery (arrowheads) as well as in the aneurysm cavity (thin arrow). Left internal carotid artery aneurysm treated with stent-assisted coiling (thick arrow) (**D**–**F**). Follow-up TOF MRA shows complete aneurysm occlusion. Postcontrast HR-VW MRI demonstrates intense contrast enhancement in the aneurysm cavity (thin arrow) with no contrast enhancement at the aneurysm periphery. Right internal carotid artery aneurysm treated with stent-assisted coiling (thick arrow) (**G**–**I**). The aneurysm is completely occluded. There is a thin rim of contrast enhancement at the aneurysm periphery (arrowheads) on postcontrast HR-VW MRI. No contrast enhancement in the aneurysm cavity is visible.

Post-procedural aneurysm contrast enhancement was observed in 30 (75.0%) aneurysms. It was interpreted as peripheral in 12 (30.0%) aneurysms, central in 9 (22.5%) aneurysms, and both peripheral and central in 9 (22.5%) aneurysms. Ten (25.0%) aneurysms did not show any contrast enhancement. The intensity of peripheral and central post-procedural aneurysm contrast enhancement was lower than or equal to that of the pituitary stalk in 95.2% and 76.5% of cases, respectively. Representative cases of post-procedural aneurysm contrast enhancement on HR-VW MRI are presented in Figure 2.

Analysis of inter-rater agreement by Cohen’s Kappa yielded the following agreement values: any kind of aneurysm enhancement, 0.94; *p* < 0.001; central aneurysm enhancement, 0.75; *p* < 0.001; peripheral aneurysm enhancement, 0.80; *p* < 0.001, respectively. In turn, analysis of inter-rater agreement by Kendall’s *W* yielded: central aneurysm enhancement intensity, 0.79; *p* < 0.001 and peripheral aneurysm enhancement intensity, 0.63; *p* < 0.001.

The statistical analysis did not reveal any relationship between time elapsed since embolization and the presence of post-procedural aneurysm contrast enhancement (x2 = 1.18; *p* = 0.277). The differences in the frequency of contrast enhancement between aneurysms with complete occlusion and those with remnant flow on follow-up MRA did not reach statistical significance (Table 3; x2 = 0.04; *p* = 0.850). There were no statistically significant differences in the frequency of contrast enhancement between aneurysms with different interval changes in the RROC (Table 3; x2 = 1.25; *p* = 0.536). Statistical analysis did not demonstrate any significant differences in the frequency of contrast enhancement between aneurysms treated with coiling and stent-assisted coiling (Table 2; x2 = 0.036; *p* = 0.850). Linear regression analysis showed that the follow-up period was not a significant predictor of more intense contrast enhancement, neither peripheral (B = −0.04; t = −0.15; *p* = 0.882; F = 0.02; *p* = 0.882) nor central (B = −0.08; t = −0.34; *p* = 0.739; F = 0.11; *p* = 0.739). Multivariate analysis did not demonstrate aneurysm size, ruptured aneurysm status, nor initial complete aneurysm occlusion to be a predictor of post-procedural aneurysm contrast enhancement on HR-VW MRI (Table 4; x2 = 6.75; *p* = 0.080).

## 4. Discussion

The aneurysm healing process following coil embolization begins with unorganized thrombus formation and progressive fibrin coverage of the coils. After a few days, inflammatory cells, macrophages, and myofibroblasts start to invade the aneurysm cavity. Eventually, the thrombus is transformed into vascularized connective tissue, of which formation progresses in a centripetal fashion. Simultaneously, the healing process at the aneurysm orifice occurs, which finally results in endothelialization of the aneurysm neck [35,36,37,38,39,40,41]. Although radiologic–histopathologic correlation studies of coiled aneurysms are missing, the vascularized connective tissue in the aneurysm cavity seems to be the most likely source of the post-procedural aneurysm contrast enhancement. According to this hypothesis, post-procedural aneurysm contrast enhancement may be the possible imaging marker of an advanced aneurysm healing stage.

In our present study, we observed contrast enhancement in the majority of the investigated post-procedural aneurysms. It was most common at the aneurysm periphery, with more than half of the aneurysms demonstrating enhancement in this location. However, we lack data on the presence of aneurysm wall contrast enhancement from the preprocedural HR-VW MRI. Over a quarter of aneurysms included in the study were ruptured. Data from the literature show that the wall enhancement is present in most of the ruptured aneurysms on HR-VW MRI. Due to this reason, peripheral contrast enhancement on post-procedural HR-VW MRI in these lesions might have been a preexisting condition in some cases. More reliable data would be obtained if HR-VW MRI was performed directly before the endovascular treatment and then at specific time intervals. We found it interesting that neither the presence nor intensity of post-procedural contrast enhancement correlated with time elapsed since embolization. This does not correspond to what we know from the histopathological analysis of the endovascularly treated aneurysms. If post-procedural aneurysm contrast enhancement represents fibrovascular tissue in the aneurysm cavity, we would expect that, over time, more aneurysms would show enhancement and enhancement would be more intense. Although we know that the aneurysm healing process progresses in a centripetal fashion, some aneurysms in our series showed contrast enhancement only in the central part of the aneurysm cavity. We suppose that the presence and distribution of contrast enhancement could possibly be related to the coil packing density. According to this, the aneurysms with the central enhancement pattern might have had higher packing density at the periphery, with larger gaps between coils loops in the central part of the aneurysm cavity. In our study, we achieved an almost perfect level of inter-rater agreement in the identification of any kind of post-procedural aneurysm contrast enhancement. The level of inter-rater agreement in the identification of peripheral and central contrast enhancement was strong and moderate, respectively. This difference could have been the result of a T1 hyperintense thrombus in the aneurysm cavity mistaken for the area of contrast enhancement.

Aneurysm wall contrast enhancement after endovascular treatment is a known imaging finding that has been investigated previously. It has been reported to be significantly correlated with aneurysm size [42,43]. Interestingly, aneurysms treated with the hydrogel-coated coils have been found to show wall contrast enhancement more frequently [43]. This can be explained by the exaggerated inflammatory response associated with this type of coil when compared to the bare platinum coils. In the paper by Fanning et al., both increasing aneurysm size and the use of hydrogel-coated coils were independent predictors of aneurysm wall contrast enhancement. On the contrary, initial and final occlusion grades as well as aneurysm recurrence were not independently associated with this imaging phenomenon. In the majority of cases, the appearance of aneurysm wall contrast enhancement has been observed to be stable over months and years after treatment [42,43].

These previous studies were based on the standard T1W images and were performed, at least partially, with a 1.5 T MRI system [42,43]. High-resolution vessel wall MRI provides several advantages over conventional MRI sequences [3]. All HR-VW MRI studies in our series were performed with a 3.0 T MRI system. A field strength of 3.0 T or higher is currently considered mandatory for vessel wall imaging, as lower field strengths (1.5 T and below) do not provide sufficient signal-to-noise ratio (SNR) and contrast-to-noise ratio (CNR) in a reasonable timely manner. In a 3.0 T MRI system, HR-VW MRI is usually acquired as a 3D FSE sequence with submillimeter resolution, which allows for the assessment of small and very small aneurysms. In our study, we acquired a 3D FSE T1W sequence with a resolution of 0.8 × 0.8 × 0.8 mm and were able to detect contrast enhancement in aneurysms as small as 3 mm. In the study by Fanning et al., 5 mm-thick axial T1W images were used which, in our view, do not allow for an accurate assessment of such small lesions [43]. Furthermore, FSE sequences are less prone to susceptibility artifacts caused by metal implants when compared to commonly used gradient echo sequences. Thus, contrast enhancement may be evaluated not only at the aneurysm periphery but also within the coil mass, which provides more imaging data for analysis. Moreover, aneurysms treated with the stent-assisted coiling may also be evaluated as images are less affected by artifacts from the neurovascular devices. The lack of studies investigating postprocedural aneurysm contrast enhancement on HR-VW MRI has been addressed in the literature recently [44,45].

Despite the use of HR-VW MRI in our series, we have not found any clinically significant correlation between post-procedural aneurysm contrast enhancement and the investigated variables. There were no statistically significant differences in the frequency of contrast enhancement between aneurysms with complete occlusion and aneurysms with remnant flow on follow-up MRA, nor between aneurysms with different interval changes in the aneurysm occlusion grade. On the contrary to Fanning et al., we did not observe increasing aneurysm size to be an independent predictor of post-procedural aneurysm contrast enhancement [43]. This discrepancy could be the result of different contrast enhancement evaluation methods as Fanning et al. investigated contrast enhancement only at the aneurysm periphery. We also included ruptured aneurysms status and initial complete aneurysm occlusion in the multivariate analysis, but they were also not predictive of contrast enhancement. Thus, based on the results of our study, the utility of this imaging sign in the prediction of aneurysm recurrence seems limited. The lack of any statistically significant relationships between post-procedural aneurysm and other variables might be due to the relatively small study sample size. For multivariate analysis, we assumed ten events per one variable as a minimum [32]. However, the study by Peduzzi et al. showed that when the number of events per variable is low, the regression coefficients may be biased in both positive and negative directions [46]. It should be also taken into consideration that despite the great capabilities of HR-VW MRI, this imaging tool might still be insufficient to accurately assess the structure of a coiled aneurysm cavity. We detected contrast enhancement in the central part of the aneurysm cavity in almost half of the cases. However, we cannot exclude the possibility that it has been obscured by the artifacts from the dense coil mass in other aneurysms. It is a known fact that neurovascular implants cause magnetic field inhomogeneity. Although fast spin echo imaging is less prone to susceptibility artifacts when compared to gradient echo imaging, some signal loss from the coil mass or stent is still inevitable. This would be a minor issue in aneurysms treated with flow diversion devices and future studies could focus on these cases.

To the best of our knowledge, there are only three published papers investigating post-procedural aneurysm contrast enhancement on HR-VW MRI [28,29,30]. The reported frequency of post-procedural aneurysm contrast enhancement on HR-VW MRI in the published papers is comparable with our study. Larsen et al. analyzed HR-VW MRI findings of 53 endovascularly treated aneurysms [28]. It is the only study which provide some evidence for clinical significance of post-procedural aneurysm contrast enhancement. They observed that wall contrast enhancement at the aneurysm neck and dome was significantly associated with time between embolization and HR-VW MRI under 6 months. On the other hand, contrast enhancement within the aneurysm cavity was significantly associated with time between embolization and HR-VW MRI longer than 6 months. In addition, contrast enhancement within the aneurysm cavity was not found in the aneurysms with reperfusion. Based on these findings, the authors concluded that contrast enhancement within the aneurysm cavity may be the imaging marker of an advanced aneurysm healing stage. They suggested that the aneurysms with this type of enhancement may not require further follow-up imaging. On the contrary, no statistically significant association between post-procedural aneurysm contrast enhancement and aneurysm recurrence was observed in our study as well as in the study by Elsheikh et al. [29]. As in our study, Elsheikh et al. did not demonstrate any relationship between post-procedural aneurysm contrast enhancement and time elapsed since embolization. Songsaeng et al. also did not find any statistically significant association [30]. Time between aneurysm embolization and HR-VW MRI varies substantially between studies, which could have led to discrepancies in their results [28,29,30]. For instance, the median follow-up period in the study by Larsen et al. was approximately three times shorter than that reported by Songsaeng et al. [28,30]. Moreover, there is a significant disproportion in the published literature between the relatively small number of investigated aneurysms and a wide range of time intervals between aneurysm embolization and HR-VW MRI. In the study by Elsheikh et al., which included only 30 aneurysms, the range was as wide as 87–5318 days [29]. In general, there are differences between published studies in the methods to evaluate post-procedural aneurysm contrast enhancement [28,29,30]. In previous studies, the intensity of post-procedural aneurysms contrast enhancement was evaluated using subjective scales. We decided to use a more quantitative approach and compared post-procedural aneurysm contrast enhancement intensity to that of pituitary stalk.

In our view, there is currently not enough evidence to support routine use of HR-VW MRI in the follow-up of patients after endovascular treatment of intracranial aneurysms. The literature in this field is scarce, with only one study reporting the possible clinical significance of this imaging finding [28]. Although the negative results of our study might be secondary to its preliminary character, they also put in doubt the clinical utility of post-procedural aneurysm contrast enhancement evaluation. It might be just a common imaging finding with no clinical meaning.

Our study has several limitations. Although comparable with other studies, small sample size could have resulted in inadequate statistical power to detect relationships between post-procedural aneurysm contrast enhancement on HR-VW MRI and investigated variables. Secondly, the size of the aneurysms included in the study varies widely. Our study sample is heterogeneous regarding endovascular treatment methods as we included aneurysms treated with coiling and stent-assisted coiling. However, only bare platinum coils were used and only one type of stent was implanted, minimizing the heterogeneity of the group. There were no other confounding factors such as inclusion of aneurysms treated with different coil types. We believe that aneurysms treated with bare platinum coils and hydrogel-coated coils should be evaluated separately since the latter are known to trigger more intense inflammatory reactions. Furthermore, the statistical analysis revealed significantly higher occlusion grades on follow-up TOF MRA in the aneurysms treated with SAC. Subgroup analysis of aneurysms treated with different endovascular techniques should be performed in future studies, but this would require a larger sample size. In our study, patients after endovascular aneurysm repair were followed up with TOF MRA. This is also our clinical practice, mainly due to the non-invasive nature of this imaging test. If a residual aneurysm is detected on TOF MRA, patients are referred for DSA, which undoubtedly remains the gold standard. In a recent meta-analysis, sensitivity and specificity of TOF MRA for the detection of residual aneurysms were 88% and 94%, respectively [47]. In a subgroup analysis of the aneurysms treated with stent-assisted coiling or flow-diversion, sensitivity was 86% and specificity was 95%. Therefore, the use of TOF MRA instead of DSA in the follow-up of the aneurysms included in our study might have had an impact on the results. In the present study, we acquired HR-VW MRI with a spatial resolution of 0.8 × 0.8 × 0.8 mm (interpolated to 0.8 × 0.8 × 0.4 mm), which is lower than the 0.5 × 0.5 × 0.5 mm recommended by the American Society of Neuroradiology [3]. On the other hand, our protocol meets the minimal HR VW-MRI sequence parameters set by the UCAN Project Investigators [48]. Both readers in our study found the quality of the images to be adequate in all of the cases. In our study, HR-VW MRI was acquired at a single time point, thus temporal changes in post-procedural aneurysm contrast enhancement were not evaluated. Furthermore, there was a wide range of time elapsed between embolization and post-procedural HR-VW MRI. Future studies should investigate temporal changes in post-procedural aneurysm contrast enhancement. We believe that observation of changes in contrast enhancement over time could possibly provide important insight on the clinical utility of this imaging finding.

Further research is needed to definitely establish whether post-procedural aneurysm contrast enhancement on HR-VW MRI has any clinical significance. If the association between this imaging finding and stable aneurysm occlusion is proven in large prospective studies, then patients with adequately occluded aneurysms demonstrating contrast enhancement could be followed up less frequently. With the increasing number of patients undergoing endovascular intracranial aneurysm repair, this will result in decreased costs for the public healthcare systems.

## 5. Conclusions

Post-procedural aneurysm contrast enhancement is a common imaging finding on HR-VW MRI. It might be distributed at the periphery or in the central part of the aneurysm cavity. No statistically significant association between post-procedural aneurysm contrast enhancement and investigated variables were found. Thus, the clinical utility of this imaging finding, especially in the prediction of aneurysm recurrence, seems limited. The results of our study do not support routine use of HR-VW MRI in the follow-up of patients after endovascular treatment of intracranial aneurysms.

## Figures and Tables

**Table 1:** Patient and aneurysm characteristics.

Characteristics	
Age (years)	52.87 (30–72)
Female (No.) (%)	32 (82.1%)
Ruptured aneurysm (No.) (%)	11 (27.5%)
Aneurysm size (mm)	6.84 (3–24)
Aneurysm location (No.) (%)	
Internal carotid artery	16 (40.0%)
Middle cerebral artery	9 (22.5%)
Anterior cerebral arteries	8 (20.0%)
Posterior communicating artery	5 (12.5%)
Posterior circulation	2 (5.0%)
The data of continuous variables are present as the mean (range).	

**Table 2:** Comparison of aneurysms treated with coiling alone vs. stent-assisted coiling.

	Coiling (*n* = 15)	Stent-Assisted Coiling (*n* = 25)
**Immediate Angiographic Outcome**		
Complete occlusion	12 (80.0%)	20 (80.0%)
Neck remnant	3 (20.0%)	5 (20.0%)
Aneurysm remnant	0 (0.0%)	0 (0.0%)
**Follow-up MR angiography**		
Complete occlusion	8 (53.3%)	17 (68.0%)
Neck remnant	2 (13.3%)	8 (32.0%)
Aneurysm remnant	5 (33.3%)	0 (0.0%)
**Interval change in aneurysm occlusion grade**		
Aneurysm recurrence	7 (46.7%)	4 (16.0%)
Stable aneurysm occlusion	7 (46.7%)	20 (80.0%)
Improved aneurysm occlusion	1 (6.7%)	1 (4.0%)
**Post-procedural aneurysm contrast enhancement**		
Yes	12 (80%)	18 (72%)
No	3 (20%)	7 (28%)

**Table 3:** Post-procedural aneurysm contrast enhancement and aneurysm occlusion grade.

**Remnant Flow on Follow-Up MRA**	**Post-Procedural Aneurysm Contrast Enhancement**	
	Yes	No
No	18 (45.0%)	7 (17.5%)
Yes	12 (30.0%)	3 (12.5%)
**Interval Change in Aneurysm Occlusion Grade**	**Post-Procedural Aneurysm Contrast Enhancement**	
	Yes	No
Aneurysm recurrence	9 (22.5%)	2 (5.0%)
Stable aneurysm occlusion	19 (47.5%)	8 (20.0%)
Improved aneurysm occlusion	2 (5.0%)	0 (0.0%)

**Table 4:** Multivariate analysis of variables independently associated with post-procedural aneurysm contrast enhancement.

Variable	Odds Ratio	95% Confidence Interval	*p*-Value
Aneurysm Size	0.67	0.42–1.06	0.089
Ruptured aneurysm	0.46	0.07–2.88	0.407
Initial complete occlusion	0.36	0.03–3.82	0.395

## Data Availability

The data presented in this study are available on request from the corresponding author.
